# Associations of serum sodium, potassium and chloride levels with the all-cause and cardiovascular diseases mortality among patients with depression

**DOI:** 10.1371/journal.pone.0314636

**Published:** 2025-02-12

**Authors:** Qingping Zeng, Siqi Jia, Yu Li, Fei She, Ping Zhang

**Affiliations:** 1 School of Clinical Medicine, Tsinghua University, Beijing, China; 2 Department of Cardiology, Beijing Tsinghua Changgung Hospital, School of Clinical Medicine, Tsinghua University, Beijing, China; 3 Department of Cardiology, Southwest Hospital, Army Medical University, Chongqing, China; 4 Dalian Medical University, Dalian, China; Tribhuvan University Institute of Medicine, NEPAL

## Abstract

**Background:**

Electrolyte disturbances are relatively common in patients with depression, but they are often overlooked, and the relationship between electrolyte changes and adverse outcomes in depression is not yet clear. This study aims to explore the impact of serum electrolyte levels on the all-cause and cardiovascular disease (CVD) mortality rates in patients with depression.

**Methods:**

This prospective cohort study included 3127 patients with depression who participated in the National Health and Nutrition Examination Survey (NHANES) from 2005 through 2018. Depression was assessed using the Patient Health Questionnaire (PHQ-9), with a PHQ-9 score ≥10 defined as depression. The data were analyzed from April 1 to July 30, 2024.

Multivariable Cox proportional hazards regression model was used to calculate the hazard ratios (HRs) and 95% confidence intervals (CIs) between serum sodium, potassium, and chloride levels and the CVD risk and all-cause mortality in patients with depression. Three multivariable models were constructed. We further stratified the analysis by age, gender, hypertension, smoking, alcohol consumption, diabetes, and drinking status. Interaction significance was estimated using P-values for the product terms between serum sodium, potassium, chloride, and stratification factors.

**Results:**

This cohort study included data from 2946 participants in the analysis (mean [SD] age, 50.13 [16.48] years; 1116 men [37.88]); During a median (IQR) follow-up of 7.2 (3.6–10.5) years, 398 deaths were recorded, of which 117 were attributed to CVD.After multivariable adjustment, compared with participants in the first quartile of serum sodium levels, the HRs of CVD mortality were 0.90(95% CI, 0.53–1.53) in the fourth quartile (p for trend = 0.484). The HRs of all-cause mortality were 0.73(95% CI, 0.55–0.99) for the fourth quartile (p for trend = 0.003). A nonlinear association was observed between serum sodium levels and all-cause mortality in patients with depression (p for overall = 0.003, p for nonlinear = 0.047). Compared with participants in the first quartile of serum potassium levels, the HRs of CVD mortality were and 1.58(95% CI, 0.98–2.54) in the fourth quartile (p for trend = 0.050), the HRs of all-cause mortality were 1.52(95% CI, 1.16–1.99) for the fourth quartile (p for trend <0.001). A nonlinear association was observed between serum potassium levels and all-cause (p for overall<0.001, p for nonlinear = 0.005) and CVD (p for nonlinear = 0.003) mortality in patients with depression. Compared with participants in the first quartile of serum chlorine levels, the HRs of CVD mortality were 0.84(95% CI, 0.49–1.46) in the fourth quartile(p for trend = 0.284). The HRs of all-cause mortality were 0.70(95% CI, 0.51–0.95) for the fourth quartile(p for trend <0.001). A nonlinear association was observed between serum chlorine levels and all-cause (p for nonlinear<0.001) and CVD (p for nonlinear<0.001) mortality in patients with depression.

**Conclusion and correlations:**

This cohort study found that in patients with depression, higher sodium is significantly correlated with lower all-cause mortality, higher potassium is significantly correlated with higher all-cause and CVD mortality, and higher chloride is significantly correlated with lower all-cause and CVD mortality.

## 1.Introduction

Depression is one of the top 25 contributors to the global burden of disease in 2019, and it is a leading cause of disability and non-fatal health impairment worldwide [1,2]. With the growth of the global population and the intensification of aging, the incidence and mortality rates of depression have shown a significant upward trend, posing a severe challenge to the global health system. The imperative for the future of holistic healthcare is to improve the emotional state of patients and the prognosis of mental and neurological disorders.

Electrolytes play a pivotal role in maintaining osmotic pressure, acid-base balance, regulating blood pressure, nerve conduction, and muscle function. Electrolyte imbalance is a common clinical phenomenon, with hyponatremia being the most prevalent [3,4]. The number of patients with depression who have comorbidities such as cardiovascular diseases (CVD), diabetes, and chronic kidney diseases (CKD) has significantly increased [5–7]. Furthermore, the likelihood of electrolyte disturbances due to the use of diuretics, antidepressant medications (such as Selective Serotonin Reuptake Inhibitors, SSRIs), neuroendocrine hormone disorders, and renal insufficiency is even greater [8–11]. Studies have indicated that a low-sodium diet has certain clinical significance in improving the prognosis of cardiovascular diseases, but research by Goldstein suggests that dietary sodium is inversely correlated with depression [12]. Numerous studies have also shown that hyponatremia may exacerbate depressive symptoms, leading to a range of adverse outcomes.

Electrolyte imbalances, including variations in serum sodium, potassium, and chloride levels, have been increasingly recognized as factors influencing mental health conditions, such as depression. Studies suggest that low potassium levels may disrupt neurotransmitter functions, which are crucial in mood regulation, potentially exacerbating depressive symptoms [13–15]. Similarly, abnormal chloride levels can influence acid-base balance and neuronal excitability, both of which may affect cognitive and emotional processing [16–18]. Besides, researchers also found the association of electrolytes and depression [19–21].

Based on these studies, this research hypothesizes that in patients with depression, reduced serum sodium levels may increase the risk of death. Utilizing data from the NHANES, this study primarily explores the correlation between all-cause and cardiovascular disease mortality and serum sodium levels in adult patients with depression.

## 2.Materials and methods

### 2.1Study population

NHANES is a nationally representative study aimed at assessing the health and nutritional status of the non-institutionalized U.S. civilian population. Conducted by the National Center for Health Statistics of the Centers for Disease Control and Prevention (CDC), NHANES has received approval from the Institutional Review Board and all participants provided written informed consent. NHANES (National Health and Nutrition Examination Survey) employs a complex, multistage probability sampling design to select participants representative of the civilian, non-institutionalized U.S. population, excluding individuals under supervised care or detention, active military personnel and their overseas family members, and any other U.S. citizens living outside the 50 states and the District of Columbia. Non-institutional group quarters, such as college dormitories, are included in the survey, with certain population subgroups oversampled to enhance the reliability and precision of health status estimates. Detailed survey manuals, consent documents, and brochures for each period are available on the NHANES website [22]. The NHANES statistical guidelines are showed in the website https://wwwn.cdc.gov/nchs/nhanes/tutorials/

The NCHS Ethics Review Board protects the rights and welfare of NHANES participants. The NHANES protocol complies with the U.S. Department of Health and Human Services Policy for the Protection of Human Research Subjects. Ethical review and approval were waived for this study as it solely used publicly available data for research and publication. Informed consent was obtained from all subjects involved in the NHANES.

**Fig 1:**
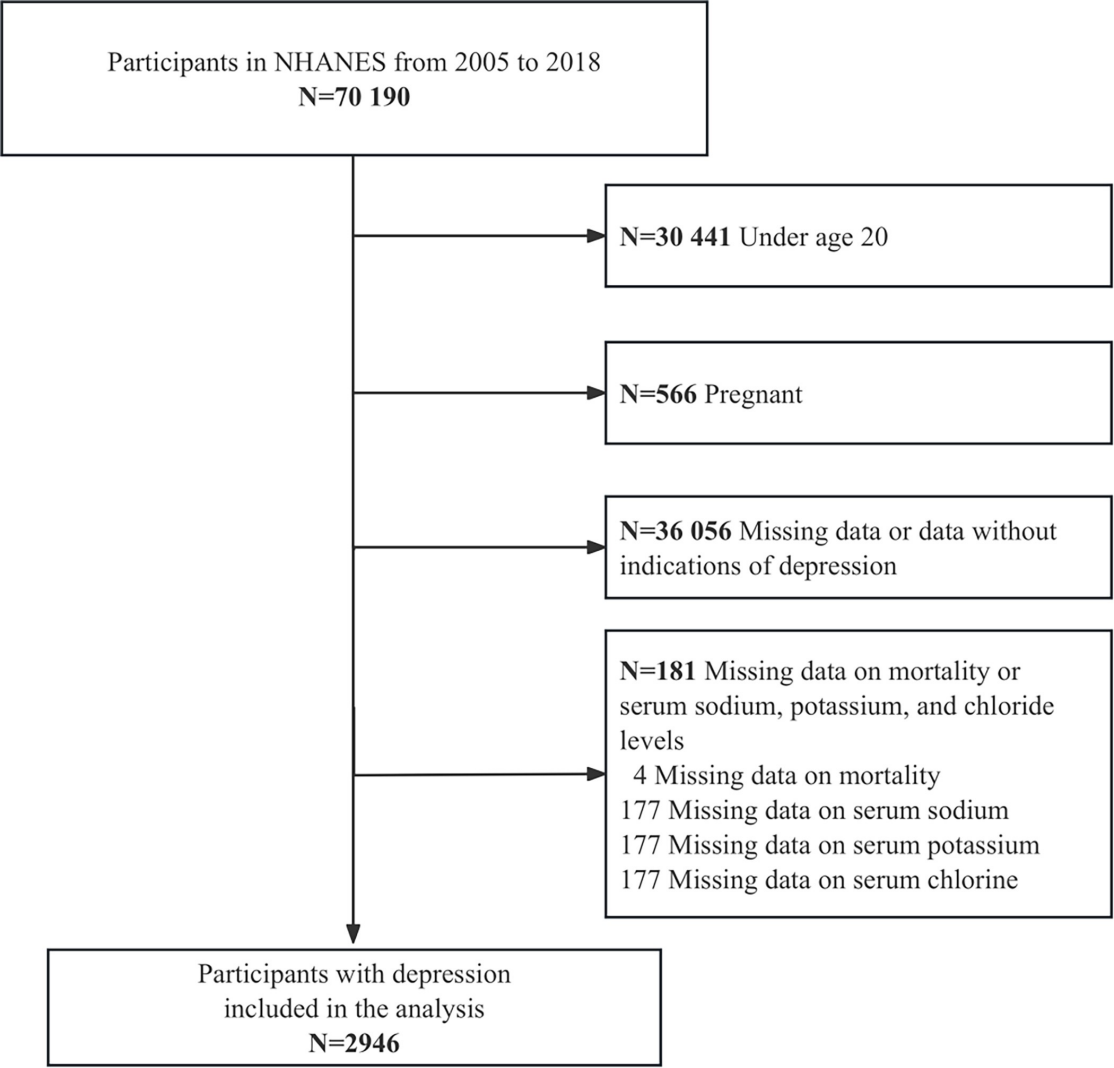
Flowchart illustrating selection of the study population in NHANES from 2005 to 2018.

This study utilized NHANES data from seven cycles spanning 2005–2018, with an initial sample size of 70,190 individuals. First, individuals under 20 years old (n = 30,441) or pregnant (n = 604) were excluded, leaving 39,183 individuals. Next, individuals without depression records (n = 30,622) or with records indicating no depression (n = 5,434) were excluded, resulting in 3,127 individuals. From this, individuals with missing mortality data (n = 4) were excluded, leaving 3,123 individuals. Finally, those with missing sodium, potassium, or chloride data (n = 177) were excluded, resulting in a final sample size of 2,946 individuals for analysis (Fig 1).

### 2.2 Depression assessment

Depression was assessed using the Patient Health Questionnaire (PHQ-9), a self-report tool based on the Diagnostic and Statistical Manual of Mental Disorders, Fourth Edition (DSM-IV) criteria for nine symptoms of depression. It evaluates depressive symptoms over the past two weeks. The PHQ-9 consists of 9 items, each scored from 0 to 3 (0 = not at all, 1 = several days, 2 = more than half the days, 3 = nearly every day), with total scores ranging from 0 to 27. According to previous studies, depression is defined as a PHQ-9 score of ≥10. This threshold, frequently used in clinical and epidemiological research, has been clinically validated with a sensitivity and specificity of 88%. Therefore, in this study, depressive symptoms were coded as "0" = no (0–9) and "1" = yes (10–27) [23].

### 2.3 Measurement of serum sodium, potassium, and chloride

Serum concentrations of sodium, potassium, and chloride were measured using standardized laboratory methods in NHANES. Serum samples from all participants were collected, processed, and stored following strict protocols to ensure sample integrity and accurate results. Specifically, serum sodium and potassium concentrations were measured using the indirect ion-selective electrode (I.S.E.) method. This technique relies on electrodes responsive to specific ions, determining ion concentrations by measuring electrolyte activity in the solution. Sodium and potassium were measured using sodium-selective and potassium-selective electrodes, respectively, with electrode voltage changes following the Nernst equation, allowing calculation of sodium and potassium concentrations in the solution. Serum chloride concentration was measured using the indirect I.S.E. method with Ag/AgCl electrodes. In this method, solid AgCl dissolves at the electrode surface until the solution reaches saturation with silver ions (Ag+) and chloride ions (Cl-). Adding chloride samples disrupts the solubility product constant (Ksp), causing AgCl to precipitate. To reestablish equilibrium, Ag+ ions are generated from the electrode tip, causing a potential change. According to the Nernst equation, this potential change is proportional to the chloride concentration in the sample.

All measurements were conducted in NHANES laboratories, adhering to stringent quality control and assurance protocols to ensure data accuracy and reliability. Detailed instructions on sample collection and handling are available on the NHANES website [24].

### 2.4 Ascertainment of mortality

The study sourced mortality causes from the NHANES Public-Use Linked Mortality Files. These files provide mortality follow-up data for NHANES participants, using the National Death Index (NDI) up to December 31, 2019. Deaths were categorized by primary cause, using the UCOD_LEADING variable to differentiate between all-cause mortality and deaths due to cardiovascular diseases, marked by UCOD_LEADING codes 001 or 005 [25].

### 2.5 Covariates

Confounding factors were assessed based on the following variables: age (years), gender (male, female), race (Mexican American, Other Hispanic, Non-Hispanic White, Non-Hispanic Black, Other Race), BMI (less than 24.9, 24.9–30, greater than 30),waist circumference (cm), serum cholesterol (mg/dL), education (lower than high school, high school, higher than high school), Poverty Income Ratio (less than 1, 1–3, greater than 3), marital status (Married, Widowed, Divorced, Separated, Never married, Living with partner), hypertension (yes or no), diabetes (yes or no), smoking (yes or no), stroke disease (yes or no),and cancer status (yes or no).

### 2.6 Statistic analysis

Categorical variables were presented as frequencies (percentages), and continuous variables were presented as medians (interquartile ranges). The Kruskal-Wallis test was used for non-normally distributed continuous variables, and the Rao-Scott second-order corrected chi-square test was used for categorical variables to compare group differences. Cox proportional hazards regression models were employed to calculate hazard ratios (HRs) and 95% confidence intervals (CIs) to examine the relationship between serum sodium, potassium, chloride levels, and the risks of all-cause mortality and cardiovascular disease (CVD) mortality in individuals with depression. The time was calculated from the NHANES interview date to the date of death or end of follow-up (December 31, 2019), whichever came first. Three models were constructed: Model 1 without any covariate adjustments; Model 2 adjusted for age, gender, race, education levels, marital status, and poverty-income ratio; and Model 3 further adjusted for diabetes, hypertension, BMI, serum cholesterol, waist circumference, smoking status, cancer, and stroke. To test linear trends, continuous variables were created by assigning median values for each category. Restricted cubic spline analysis with four knots (at the 5th, 35th, 65th, and 95th percentiles) was used to investigate nonlinear relationships between serum sodium, potassium, chloride, and all-cause and CVD mortality, with nonlinearity assessed via the likelihood ratio test.

We further stratified the analysis by age, gender, hypertension, smoking, alcohol consumption, diabetes, and drinking status. Interaction significance was estimated using P-values for the product terms between serum sodium, potassium, chloride, and stratification factors.

All statistical analyses were performed using R software, version 4.2.3 (R Project for Statistical Computing), from April 1 to July 30, 2024. A two-sided P-value of < .05 was considered the threshold for statistical significance.

## 3.Results

### 3.1 Characteristic

A total of 2946 confirmed cases of depression were included in this study (mean [SD] age, 50.13 [16.48] years; 1116 men [37.88] and 1830 women [62.12%]; The baseline characteristics of 2946 participants with depression according to quartile of serum electrolyte (serum sodium: 1067 participants in quartile 1 (<138 mmol/L); 520 participants in quartile 2 (138–139 mmol/L); 851 quartile 3 (139–141 mmol/L); and 508 quartile 4 (>141 mmol/L). serum potassium: 1010 participants in quartile 1 (<3.8 mmol/L); 729 participants in quartile 2 (3.8–4.0 mmol/L); 571 quartile 3 (4.0–4.2 mmol/L); and 636 quartile 4 (>4.2 mmol/L). serum chlorine: 774 participants in quartile 1 (<102.0 mmol/L); 1056 participants in quartile 2 (102.0–103.8 mmol/L); 675 quartile 3 (103.8–105.0 mmol/L); and 441 quartile 4 (>105.0 mmol/L)) are summarized in Table 1.

**Table 1:** Baseline characteristics of participants with depression by serum sodium, potassium and chlorine levels in NHANES 2005–2018.

Characteristic	Participants, No. (%) Serum sodium level, mmol/mL						Serum potassium level, mmol/mL					Serum chlorine level, mmol/mL				
	Total	Quartile 1	Quartile 2	Quartile 3	Quartile 4	P	Quartile 1	Quartile 2	Quartile 3	Quartile 4	P	Quartile 1	Quartile 2	Quartile 3	Quartile 4	P
	2946	1067	520	851	508		1010	729	571	636		774	1056	675	441	
age, Mean (SD)	50.13 (16.48)	48.99 (16.06)	46.98 (16.37)	51.21 (16.61)	53.93 (16.39)	< .001	47.35 (16.01)	48.11 (16.15)	50.70 (16.44)	56.34 (15.95)	< .001	53.51 (15.98)	49.14 (16.23)	48.69 (16.54)	48.75 (17.05)	< .001
sex, n(%)						0.373					< .001					< .001
Female	1830 (62.12)	671 (62.89)	307 (59.04)	527 (61.93)	325 (63.98)		694 (68.71)	470 (64.47)	345 (60.42)	321 (50.47)		425 (54.91)	642 (60.80)	439 (65.04)	324 (73.47)	
Male	1116 (37.88)	396 (37.11)	213 (40.96)	324 (38.07)	183 (36.02)		316 (31.29)	259 (35.53)	226 (39.58)	315 (49.53)		349 (45.09)	414 (39.20)	236 (34.96)	117 (26.53)	
race/ethnicity, n(%)						0.030					< .001					< .001
Mexican American	450 (15.27)	137 (12.84)	85 (16.35)	152 (17.86)	76 (14.96)		159 (15.74)	116 (15.91)	92 (16.11)	83 (13.05)		80 (10.34)	163 (15.44)	126 (18.67)	81 (18.37)	
Other Hispanic	377 (12.80)	139 (13.03)	72 (13.85)	114 (13.40)	52 (10.24)		125 (12.38)	99 (13.58)	68 (11.91)	85 (13.36)		94 (12.14)	143 (13.54)	81 (12.00)	59 (13.38)	
Non-Hispanic White	1257 (42.67)	491 (46.02)	219 (42.12)	341 (40.07)	206 (40.55)		366 (36.24)	324 (44.44)	259 (45.36)	308 (48.43)		398 (51.42)	430 (40.72)	275 (40.74)	154 (34.92)	
Non-Hispanic Black	638 (21.66)	224 (20.99)	109 (20.96)	181 (21.27)	124 (24.41)		271 (26.83)	143 (19.62)	107 (18.74)	117 (18.40)		141 (18.22)	232 (21.97)	140 (20.74)	125 (28.34)	
Other Race	224 (7.60)	76 (7.12)	35 (6.73)	63 (7.40)	50 (9.84)		89 (8.81)	47 (6.45)	45 (7.88)	43 (6.76)		61 (7.88)	88 (8.33)	53 (7.85)	22 (4.99)	
hypertension, n(%)						< .001					< .001					< .001
No	1147 (38.93)	402 (37.68)	238 (45.77)	338 (39.72)	169 (33.27)		405 (40.10)	304 (41.70)	239 (41.86)	199 (31.29)		218 (28.17)	418 (39.58)	307 (45.48)	204 (46.26)	
Yes	1799 (61.07)	665 (62.32)	282 (54.23)	513 (60.28)	339 (66.73)		605 (59.90)	425 (58.30)	332 (58.14)	437 (68.71)		556 (71.83)	638 (60.42)	368 (54.52)	237 (53.74)	
PAQ, n(%)						0.052*					0.062*					0.311*
No	1931 (65.55)	696 (65.23)	327 (62.88)	568 (66.75)	340 (66.93)		659 (65.25)	480 (65.84)	355 (62.17)	437 (68.71)		503 (64.99)	690 (65.34)	442 (65.48)	296 (67.12)	
Yes	991 (33.64)	355 (33.27)	192 (36.92)	278 (32.67)	166 (32.68)		345 (34.16)	246 (33.74)	205 (35.90)	195 (30.66)		259 (33.46)	358 (33.90)	230 (34.07)	144 (32.65)	
Unable to do activity	21 (0.71)	14 (1.31)	0 (0.00)	5 (0.59)	2 (0.39)		5 (0.50)	3 (0.41)	9 (1.58)	4 (0.63)		10 (1.29)	8 (0.76)	2 (0.30)	1 (0.23)	
Missing	3 (0.10)	2 (0.19)	1 (0.19)	0 (0.00)	0 (0.00)		1 (0.10)	0 (0.00)	2 (0.35)	0 (0.00)		2 (0.26)	0 (0.00)	1 (0.15)	0 (0.00)	
Educational, n(%)						0.152*					0.397*					0.911*
lower than high school	704 (23.90)	255 (23.90)	120 (23.08)	191 (22.44)	138 (27.17)		244 (24.16)	175 (24.01)	137 (23.99)	148 (23.27)		188 (24.29)	253 (23.96)	158 (23.41)	105 (23.81)	
high school	1062 (36.05)	398 (37.30)	179 (34.42)	322 (37.84)	163 (32.09)		348 (34.46)	255 (34.98)	207 (36.25)	252 (39.62)		274 (35.40)	374 (35.42)	243 (36.00)	171 (38.78)	
higher than high school	1178 (39.99)	414 (38.80)	220 (42.31)	338 (39.72)	206 (40.55)		418 (41.39)	299 (41.02)	226 (39.58)	235 (36.95)		311 (40.18)	429 (40.62)	273 (40.44)	165 (37.41)	
Missing	2 (0.07)	0 (0.00)	1 (0.19)	0 (0.00)	1 (0.20)		0 (0.00)	0 (0.00)	1 (0.18)	1 (0.16)		1 (0.13)	0 (0.00)	1 (0.15)	0 (0.00)	
diabetes, n(%)						< .001					< .001					< .001
No	2226 (75.56)	732 (68.60)	409 (78.65)	683 (80.26)	402 (79.13)		820 (81.19)	579 (79.42)	424 (74.26)	403 (63.36)		506 (65.37)	800 (75.76)	559 (82.81)	361 (81.86)	
Yes	720 (24.44)	335 (31.40)	111 (21.35)	168 (19.74)	106 (20.87)		190 (18.81)	150 (20.58)	147 (25.74)	233 (36.64)		268 (34.63)	256 (24.24)	116 (17.19)	80 (18.14)	
smoking, n(%)						0.077					0.221					0.020*
No	1191 (40.43)	401 (37.58)	224 (43.08)	357 (41.95)	209 (41.14)		423 (41.88)	289 (39.64)	240 (42.03)	239 (37.58)		281 (36.30)	429 (40.62)	290 (42.96)	191 (43.31)	
Yes	1754 (59.54)	666 (62.42)	296 (56.92)	494 (58.05)	298 (58.66)		587 (58.12)	440 (60.36)	330 (57.79)	397 (62.42)		493 (63.70)	627 (59.38)	385 (57.04)	249 (56.46)	
Missing	1 (0.03)	0 (0.00)	0 (0.00)	0 (0.00)	1 (0.20)		0 (0.00)	0 (0.00)	1 (0.18)	0 (0.00)		0 (0.00)	0 (0.00)	0 (0.00)	1 (0.23)	
cancer or malignancy, n(%)						0.040*					0.460*					0.700*
No	2604 (88.39)	955 (89.50)	469 (90.19)	750 (88.13)	430 (84.65)		896 (88.71)	650 (89.16)	510 (89.32)	548 (86.16)		677 (87.47)	937 (88.73)	601 (89.04)	389 (88.21)	
Yes	339 (11.51)	111 (10.40)	50 (9.62)	100 (11.75)	78 (15.35)		113 (11.19)	78 (10.70)	61 (10.68)	87 (13.68)		96 (12.40)	119 (11.27)	73 (10.81)	51 (11.56)	
Missing	3 (0.10)	1 (0.09)	1 (0.19)	1 (0.12)	0 (0.00)		1 (0.10)	1 (0.14)	0 (0.00)	1 (0.16)		1 (0.13)	0 (0.00)	1 (0.15)	1 (0.23)	
congestive heart failure, n(%)						0.125*					< .001*					0.003*
No	2724 (92.46)	972 (91.10)	493 (94.81)	795 (93.42)	464 (91.34)		947 (93.76)	685 (93.96)	531 (92.99)	561 (88.21)		691 (89.28)	980 (92.80)	640 (94.81)	413 (93.65)	
Yes	208 (7.06)	88 (8.25)	26 (5.00)	53 (6.23)	41 (8.07)		58 (5.74)	39 (5.35)	39 (6.83)	72 (11.32)		75 (9.69)	72 (6.82)	33 (4.89)	28 (6.35)	
Missing	14 (0.48)	7 (0.66)	1 (0.19)	3 (0.35)	3 (0.59)		5 (0.50)	5 (0.69)	1 (0.18)	3 (0.47)		8 (1.03)	4 (0.38)	2 (0.30)	0 (0.00)	
stroke, n(%)						0.237*					0.003*					0.016*
No	2698 (91.58)	970 (90.91)	488 (93.85)	780 (91.66)	460 (90.55)		924 (91.49)	688 (94.38)	525 (91.94)	561 (88.21)		689 (89.02)	966 (91.48)	630 (93.33)	413 (93.65)	
Yes	237 (8.04)	91 (8.53)	30 (5.77)	70 (8.23)	46 (9.06)		83 (8.22)	40 (5.49)	44 (7.71)	70 (11.01)		79 (10.21)	88 (8.33)	42 (6.22)	28 (6.35)	
Missing	11 (0.37)	6 (0.56)	2 (0.38)	1 (0.12)	2 (0.39)		3 (0.30)	1 (0.14)	2 (0.35)	5 (0.79)		6 (0.78)	2 (0.19)	3 (0.44)	0 (0.00)	
PIR, n(%)						0.339					0.001					0.966
< 1	1114 (37.81)	415 (38.89)	203 (39.04)	316 (37.13)	180 (35.43)		406 (40.20)	302 (41.43)	174 (30.47)	232 (36.48)		291 (37.60)	396 (37.50)	256 (37.93)	171 (38.78)	
1–3	1331 (45.18)	487 (45.64)	225 (43.27)	374 (43.95)	245 (48.23)		442 (43.76)	299 (41.02)	291 (50.96)	299 (47.01)		356 (45.99)	473 (44.79)	301 (44.59)	201 (45.58)	
>3	501 (17.01)	165 (15.46)	92 (17.69)	161 (18.92)	83 (16.34)		162 (16.04)	128 (17.56)	106 (18.56)	105 (16.51)		127 (16.41)	187 (17.71)	118 (17.48)	69 (15.65)	
Marital Status, n(%)						< .001					0.003					0.004
Married	1060 (35.98)	391 (36.64)	195 (37.50)	312 (36.66)	162 (31.89)		344 (34.06)	250 (34.29)	211 (36.95)	255 (40.09)		284 (36.69)	364 (34.47)	265 (39.26)	147 (33.33)	
Widowed	298 (10.12)	100 (9.37)	39 (7.50)	94 (11.05)	65 (12.80)		83 (8.22)	67 (9.19)	65 (11.38)	83 (13.05)		97 (12.53)	94 (8.90)	58 (8.59)	49 (11.11)	
Divorced	516 (17.52)	166 (15.56)	72 (13.85)	168 (19.74)	110 (21.65)		178 (17.62)	134 (18.38)	89 (15.59)	115 (18.08)		148 (19.12)	194 (18.37)	102 (15.11)	72 (16.33)	
Separated	201 (6.82)	73 (6.84)	38 (7.31)	52 (6.11)	38 (7.48)		73 (7.23)	53 (7.27)	32 (5.60)	43 (6.76)		52 (6.72)	72 (6.82)	41 (6.07)	36 (8.16)	
Never married	590 (20.03)	233 (21.84)	122 (23.46)	146 (17.16)	89 (17.52)		230 (22.77)	156 (21.40)	114 (19.96)	90 (14.15)		148 (19.12)	221 (20.93)	127 (18.81)	94 (21.32)	
Living with partner	278 (9.44)	104 (9.75)	53 (10.19)	79 (9.28)	42 (8.27)		100 (9.90)	69 (9.47)	59 (10.33)	50 (7.86)		45 (5.81)	109 (10.32)	81 (12.00)	43 (9.75)	
Missing	3 (0.10)	0 (0.00)	1 (0.19)	0 (0.00)	2 (0.39)		2 (0.20)	0 (0.00)	1 (0.18)	0 (0.00)		0 (0.00)	2 (0.19)	1 (0.15)	0 (0.00)	
BMI, Mean(SD)	30.96 (8.15)	31.70 (8.77)	30.45 (7.38)	30.76 (7.98)	30.25 (7.74)	0.002	30.42 (8.19)	31.41 (8.11)	31.23 (8.37)	31.03 (7.93)	0.063	31.25 (9.10)	30.71 (7.75)	30.66 (7.83)	31.50 (7.80)	0.184
WC, Mean(SD)	103.56 (18.30)	104.98 (19.07)	102.09 (17.50)	103.36 (18.07)	102.41 (17.66)	0.008	101.26 (18.40)	104.36 (17.70)	104.44 (18.51)	105.49 (18.29)	< .001	105.03 (19.69)	102.80 (17.97)	102.33 (17.73)	104.68 (17.20)	0.009
Total cholesterol (mmol/L), Mean(SD)	5.03 (1.15)	5.10 (1.21)	4.97 (1.14)	5.03 (1.12)	4.95 (1.08)	0.047	5.01 (1.10)	5.12 (1.15)	5.12 (1.21)	4.88 (1.16)	< .001	5.17 (1.30)	5.07 (1.14)	4.94 (1.04)	4.84 (1.04)	< .001
Sodium (mmol/L), Mean (SD)	139.23 (2.62)	136.63 (1.86)	139.00 (0.00)	140.46 (0.50))	142.88 (1.41)	< .001	139.24 (2.50)	139.04 (2.44)	139.36 (2.47)	139.32 (3.08)	0.116	137.67 (3.02)	139.27 (2.14)	140.05 (2.02)	140.62 (2.34)	< .001
Potassium (mmol/L), Mean (SD)	3.99 (0.36)	3.99 (0.35)	3.97 (0.35)	3.99 (0.36)	4.01 (0.41)	0.445	3.62 (0.19)	3.94 (0.06)	4.14 (0.05)	4.49 (0.24)	< .001	3.99 (0.41)	3.98 (0.35)	3.98 (0.34)	4.01 (0.36)	0.507
Chloride (mmol/L), Mean (SD)	103.29 (3.37)	101.48 (3.42)	103.60 (2.91)	104.47 (2.63)	104.78 (3.09)	< .001	103.36 (3.37)	103.42 (3.15	103.39 (3.25)	102.93 (3.69)	0.027	99.00 (2.34)	103.05 (0.82)	105.45 (0.50)	108.07 (1.36)	< .001
eGFR, Mean (SD)	92.57 (24.96)	93.92 (26.35)	95.67 (23.11)	91.50 (24.14)	88.38 (24.52)	< .001	96.63 (23.10)	96.71 (23.02	91.81 (23.50)	82.08 (27.94)	< .001	88.57 (26.04)	93.53 (23.86)	95.56 (23.14)	92.72 (27.35)	< .001
Phosphorus (mmol/L), Mean (SD)	1.21 (0.20)	1.21 (0.19)	1.21 (0.20)	1.22 (0.20)	1.22 (0.22)	0.698	1.19 (0.19)	1.21 (0.18	1.22 (0.19)	1.24 (0.23)	< .001	1.23 (0.21)	1.21 (0.19)	1.20 (0.19)	1.21 (0.19)	0.027
Total Calcium (mmol/L), Mean (SD)	2.34 (0.10)	2.33 (0.09)	2.34 (0.09)	2.35 (0.10)	2.35 (0.10)	< .001	2.33 (0.09)	2.34 (0.09)	2.36 (0.10)	2.35 (0.10)	< .001	2.36 (0.10)	2.35 (0.09)	2.34 (0.09)	2.32 (0.10)	< .001
Bicarbonate (mmol/L), Mean (SD)	24.95 (2.50)	24.21 (2.41)	24.75 (2.19)	25.25 (2.32)	26.19 (2.68)	< .001	24.95 (2.50)	24.78 (2.40)	24.92 (2.41)	25.17 (2.65)	0.043	26.01 (2.71)	25.16 (2.14)	24.49 (2.14)	23.28 (2.36)	< .001

a: All estimates accounted for complex survey designs, and all percentages were weighted.

*: Simulated p-value.

Abbreviations:PAQ, Physical activity; PIR, poverty income ratio;BMI, body mass index; WC, waist circumference.

Compared with participants in the first quartile of serum sodium levels, participants with higher serum sodium level were older, had higher levels of serum total calciumhad,bicarbonate, had lower BMI, waist circumference, serum total cholesterol and eGFR, more often Non-Hispanic, cancer or malignancy, hypertension, without diabetes. Similarities were found in the percentages of physical activity, sex, education, smoking status, congestive heart failure, stroke, poverty income ratio, serum phosphorus levels among the 4 groups. Compared with participants in the first quartile of serum potassium levels, participants with higher serum sodium level were older, had higher levels of serum phosphorus, total calciumhad and bicarbonate, had lower waist circumference, serum total cholesterol and eGFR, more often female, Non-Hispanic, congestive heart failure, stroke, hypertension, diabetes. Similarities were found in the percentages of physical activity, education, smoking status, cancer or malignancy, BMI levels among the 4 groups. Compared with participants in the first quartile of serum chlorine levels, participants with higher serum sodium level were younger, had higher levels of serum total calciumhad and eGFR, had lower waist circumference and serum total cholesterol, more less hypertension, diabetes, congestive heart failure, stroke, smoking, more often female, Non-Hispanic. Similarities were found in the percentages of physical activity, education, BMI, poverty income ratio, cancer or malignancy, serum phosphorus levels among the 4 groups.

### 3.2 Serum sodium and mortality

**Fig 2:**
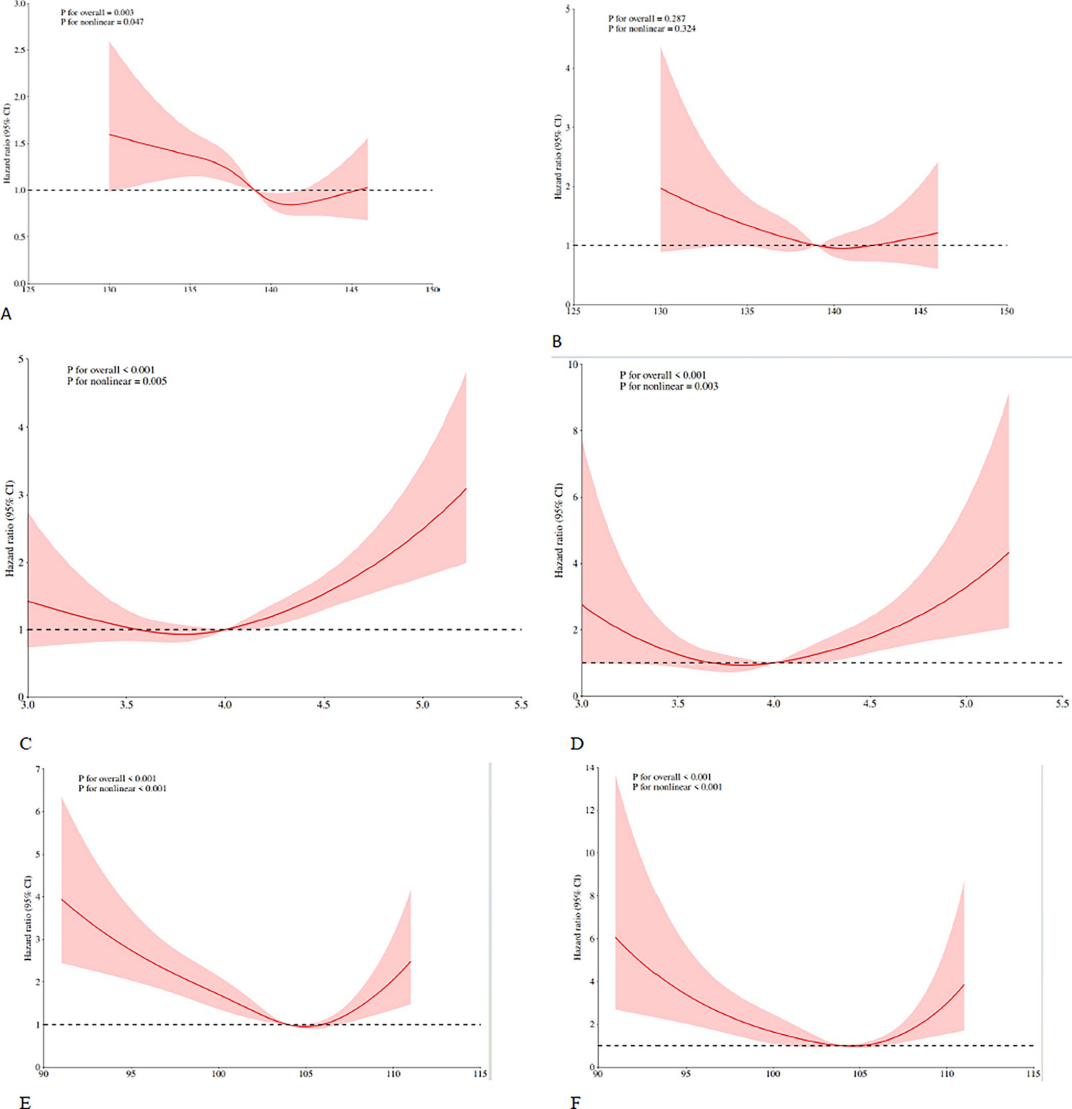
Association of serum sodium, potassium and chloride levels with all-cause and CVD mortality among adults with depression in the NHANES 2005–2018. Restricted cubic spline fitting for the association between sodium, potassium, and chlorine serum Levels with mortality. The association of serum sodium Levels with the all-cause (A) and cardiovascular diseases (CVD) (B) mortality. The association of serum potassium Levels with the all-cause (C) and cardiovascular diseases (CVD) (D) mortality. The association of serum chlorine Levels with the all-cause (E) and cardiovascular diseases (CVD) (F) mortality.

During a median (IQR) follow-up of 7.2 (3.6–10.5) years, 398 deaths were recorded, of which 117 were attributed to CVD. After multivariable adjustment, compared with the reference group (the first quartile), the HRs of CVD mortality were 0.67 (95% CI, 0.36–1.24) in the second quartile, 0.78(95% CI, 0.50–1.22) in the third quartile, and 0.90(95% CI, 0.53–1.53) in the fourth quartile (p for trend = 0.484) (Table 2). Similarly, compared with the reference group (quartile 1), the HRs of all-cause mortality were 0.69(95% CI, 0.50–0.94) for the second quartile, 0.66(95% CI, 0.51–0.84) for the third quartile, and 0.73(95% CI, 0.55–0.99) for the fourth quartile (p for trend = 0.003). A nonlinear association was observed between serum sodium levels and all-cause mortality in patients with depression(p for overall = 0.003, p for nonlinear = 0.047) (Table 2, Fig 2A). No significant association was observed between serum sodium levels and CVD mortality in patients with depression (p for overall = 0.287, p for nonlinear = 0.324) (Fig 2B).

**Table 2:** Hazard ratios of CVD and all-cause mortality by serum sodium, potassium and chlorine levels among adults with depression in NHANES 2005–2018.

Variable	Hazard ratio (95% CI)				p for trend
	Quartile 1	Quartile 2	Quartile 3	Quartile 4	
**All-cause mortality**					
**Serum sodium**					
Model 1^a^	1 [Reference]	0.61(0.45, 0.83)	0.79(0.62, 1.00)	0.98(0.74, 1.30)	0.430
Model 2^b^	1 [Reference]	0.69(0.51, 0.95)	0.66(0.51, 0.84)	0.70(0.53, 0.94)	0.001
Model 3^c^	1 [Reference]	0.69(0.50, 0.94)	0.66(0.51, 0.84)	0.73(0.55, 0.99)	0.003
**Serum potassium**					
Model 1^a^	1 [Reference]	0.95(0.70, 1.30)	1.82(1.37, 2.42)	2.96(2.29, 3.83)	<0.001
Model 2^b^	1 [Reference]	0.92(0.67, 1.25)	1.25(0.93, 1.68)	1.57(1.20, 2.05)	<0.001
Model 3^c^	1 [Reference]	0.92(0.67, 1.26)	1.25(0.93, 1.68)	1.52(1.16, 1.99)	<0.001
**Serum chlorine**					
Model 1^a^	1 [Reference]	0.53(0.42, 0.68)	0.35(0.26, 0.47)	0.52(0.39, 0.71)	<0.001
Model 2^b^	1 [Reference]	0.66(0.52, 0.83)	0.50(0.37, 0.67)	0.69(0.51, 0.93)	<0.001
Model 3^c^	1 [Reference]	0.65(0.51, 0.82)	0.50(0.37, 0.68)	0.70(0.51, 0.95)	<0.001
**CVD mortality**					
**Serum sodium**					
Model 1^a^	1 [Reference]	0.54(0.29, 0.99)	0.93(0.60, 1.43)	1.11(0.67, 1.86)	0.724
Model 2^b^	1 [Reference]	0.67(0.36, 1.24)	0.75(0.48, 1.16)	0.78(0.46, 1.31)	0.234
Model 3^c^	1 [Reference]	0.67(0.36, 1.24)	0.78(0.50, 1.22)	0.90(0.53, 1.53)	0.484
**Serum potassium**					
Model 1^a^	1 [Reference]	0.92(0.52, 1.63)	1.45(0.84, 2.52)	3.20(2.02, 5.07)	<0.001
Model 2^b^	1 [Reference]	0.92(0.51, 1.64)	1.01(0.57, 1.80)	1.70(1.06, 2.75)	0.022
Model 3^c^	1 [Reference]	0.92(0.51, 1.65)	1.0(0.56, 1.75)	1.58(0.98, 2.54)	0.050
**Serum chlorine**					
Model 1^a^	1 [Reference]	0.46(0.29, 0.71)	0.31(0.18, 0.55)	0.58(0.35, 0.98)	0.006
Model 2^b^	1 [Reference]	0.56(0.36, 0.88)	0.47(0.27, 0.83)	0.73(0.43, 1.25)	0.101
Model 3^c^	1 [Reference]	0.56(0.36, 0.88)	0.52(0.29, 0.92)	0.84(0.49, 1.46)	0.284

Abbreviations: CI, Confidence Interval, CVD, Cardiovascular disease; PIR, poverty income ratio; BMI, body mass index; WC, waist circumference.

Model 1: Not adjusted.

Model 2: Adjusted by age, sex, race/ethnicity, PIR, education, Marital status.

Model 3: Adjusted by age, sex, race/ethnicity, PIR, education, Marital status, smoking status, drinking status, BMI, WC, hypertension, hyperlipidemia, Total cholesterol, stroke, smoking, cancer or malignancy, and diabetes.

### 3.3 Serum potassium and mortality

After multivariable adjustment, compared with the reference group (the first quartile), the HRs of CVD mortality were 0.92(95% CI, 0.51–1.65) in the second quartile, 1.0(95% CI, 0.56–1.75) in the third quartile, and 1.58(95% CI, 0.98–2.54) in the fourth quartile (p for trend = 0.050) (Table 2). Similarly, compared with the reference group (quartile 1), the HRs of all-cause mortality were 0.92(95% CI, 0.67, 1.26) for the second quartile, 1.25(95% CI, 0.93–1.68) for the third quartile, and 1.52(95% CI, 1.16–1.99) for the fourth quartile (p for trend <0.001). A nonlinear association was observed between serum potassium levels and all-cause mortality in patients with depression (p for overall<0.001, p for nonlinear = 0.005) (Table 2, Fig 2C). A nonlinear association was observed between serum potassium levels and CVD mortality in patients with depression (p for overall<0.001, p for nonlinear = 0.003) (Fig 2D).

### 3.4 Serum chlorine and mortality

After multivariable adjustment, compared with the reference group (the first quartile), the HRs of CVD mortality were 0.56(95% CI, 0.36–0.88) in the second quartile, 0.52(95% CI, 0.29–0.92) in the third quartile, and 0.84(95% CI, 0.49–1.46) in the fourth quartile (p for trend = 0.284) (Table 2). Similarly, compared with the reference group (quartile 1), the HRs of all-cause mortality were 0.65(95% CI, 0.51–0.82) for the second quartile, 0.50(95% CI, 0.37–0.68) for the third quartile, and 0.70(95% CI, 0.51–0.95) for the fourth quartile (p for trend <0.001). A nonlinear association was observed between serum chlorine levels and all-cause mortality in patients with depression (p for overall<0.001, p for nonlinear<0.001) (Table 2, Fig 2E). A nonlinear association was observed between serum chlorine levels and CVD mortality in patients with depression (p for overall<0.001, p for nonlinear<0.001) (Fig 2F).

### 3.5 Stratified and sensitivity analyses

We found a significant interaction between serum sodium and economic levels with the risk of all-cause mortality in patients with depression (P  = 0.04 for interaction). In the subgroup with PIR<1, compared with the reference group (the first quartile), the HR for all-cause mortality in the second, third, and fourth quartile was 0.52(95% CI, 0.31–0.88), 0.53(95% CI, 0.34–0.80), and 0.53(95% CI, 0.33–0.87), respectively. In the subgroup with PIR 2~3, compared with the reference group, the HR for all-cause mortality in the second, third, and fourth quartile was 0.84(95% CI, 0.53–1.34), 0.59(95% CI, 0.41–0.85), and 0.65(95% CI, 0.41–1.02), respectively. In the subgroup with PIR>3, compared with the reference group, the HR for all-cause mortality in the second, third, and fourth quartile was 0.82(95% CI, 0.33–2.05), 1.99(95% CI, 0.94–4.19), and 2.61(95% CI, 1.10–6.17), respectively. However, no significant interactions were found between serum sodium and any other strata variables with the risk of all-cause mortality (Table 3). There were no significant interactions between serum sodium and any strata variables with the risk of CVD mortality (Table 3).

**Table 3:** Associations of serum sodium, potassium, and chlorine levels with all-cause and CVD mortality in various subgroups among adults with depression in NHANES 2005–2018.

Variable	Hazard ratio (95% CIs) by quartile^a^				p for interaction
	Quartile 1	Quartile 2	Quartile 3	Quartile 4	
**All-cause mortality**					
**Serum sodium**					
sex					
Female	1 [Reference]	0.74(0.46, 1.18)	0.73(0.52, 1.04)	0.67(0.44, 1.01)	0.97
Male	1 [Reference]	0.56(0.36, 0.86)	0.56(0.39, 0.81)	0.75(0.49, 1.16)	
age					
<65	1 [Reference]	0.69(0.45, 1.06)	0.82(0.58, 1.16)	0.66(0.41, 1.06)	0.11
≥65	1 [Reference]	0.76(0.47, 1.22)	0.66(0.46, 0.94)	1.03(0.69, 1.53)	
hypertension					
No	1 [Reference]	0.76(0.41, 1.43)	0.72(0.39, 1.31)	0.52(0.24, 1.12)	0.91
Yes	1 [Reference]	0.64(0.44, 0.93)	0.65(0.50, 0.86)	0.77(0.55, 1.06)	
diabetes					
No	1 [Reference]	0.75(0.49, 1.15)	0.59(0.43, 0.82)	0.68(0.47, 1.00)	0.51
Yes	1 [Reference]	0.65(0.40, 1.06)	0.79(0.53, 1.17)	0.78(0.48, 1.28)	
PIR					
<1	1 [Reference]	0.52(0.31, 0.88)	0.53(0.34, 0.80)	0.53(0.33, 0.87)	0.04
1–3	1 [Reference]	0.84(0.53, 1.34)	0.59(0.41, 0.85)	0.65(0.41, 1.02)	
>3	1 [Reference]	0.82(0.33, 2.05)	1.99(0.94, 4.19)	2.61(1.10, 6.17)	
BMI (kg/m 2)					
<24.9	1 [Reference]	0.75(0.41, 1.39)	0.53(0.32, 0.87)	0.87(0.51, 1.50)	0.72
25.0–29.9	1 [Reference]	0.66(0.35, 1.26)	0.59(0.35, 1.01)	0.62(0.32, 1.20)	
>30.0	1 [Reference]	0.70(0.44, 1.10)	0.81(0.56, 1.16)	0.76(0.48, 1.19)	
**Serum potassium**					
sex					
Female	1 [Reference]	1.1(0.73, 1.68)	1.42(0.94, 2.13)	1.49(1.01, 2.18)	0.92
Male	1 [Reference]	0.76(0.46, 1.25)	1.22(0.78, 1.89)	1.51(1.03, 2.22)	
age					
<65	1 [Reference]	1.06(0.69, 1.61)	1.73(1.15, 2.60)	2.10(1.43, 3.08)	0.27
≥65	1 [Reference]	0.80(0.49, 1.31)	1.14(0.74, 1.78)	1.60(1.09, 2.36)	
hypertension					
No	1 [Reference]	1.67(0.79, 3.55)	2.02(0.98, 4.16)	2.62(1.30, 5.31)	0.03
Yes	1 [Reference]	0.81(0.57, 1.16)	1.21(0.87, 1.68)	1.39(1.04, 1.87)	
diabetes					
No	1 [Reference]	1.06(0.71, 1.57)	1.31(0.89, 1.95)	1.56(1.08, 2.26)	0.69
Yes	1 [Reference]	0.76(0.44, 1.32)	1.33(0.84, 2.12)	1.46(0.98, 2.19)	
PIR					
<1	1 [Reference]	1.05(0.66, 1.69)	1.64(1.01, 2.65)	1.28(0.83, 1.98)	0.24
1–3	1 [Reference]	0.89(0.54, 1.45)	1.06(0.68, 1.65)	1.78(1.20, 2.64)	
>3	1 [Reference]	0.67(0.26, 1.71)	1.68(0.73, 3.88)	1.42(0.63, 3.20)	
BMI (kg/m 2)					
<24.9	1 [Reference]	1.01(0.57, 1.81)	1.08(0.62, 1.87)	1.83(1.11, 3.02)	0,47
25.0–29.9	1 [Reference]	0.75(0.38, 1.47)	1.32(0.70, 2.48)	1.45(0.83, 2.53)	
>30.0	1 [Reference]	0.85(0.53, 1.37)	1.88(1.23, 2.89)	1.80(1.20, 2.68)	
**Serum chlorine**					
sex					
Female	1 [Reference]	0.62(0.44, 0.88)	0.41(0.26, 0.65)	0.68(0.44, 1.03)	0.88
Male	1 [Reference]	0.68(0.48, 0.97)	0.60(0.40, 0.92)	0.70(0.43, 1.14)	
age					
<65	1 [Reference]	0.63(0.45, 0.89)	0.37(0.23, 0.59)	0.60(0.38, 0.94)	0.10
≥65	1 [Reference]	0.67(0.47, 0.96)	0.72(0.47, 1.10)	1.19(0.76, 1.85)	
hypertension					
No	1 [Reference]	0.73(0.42, 1.29)	0.62(0.31, 1.26)	0.43(0.19, 0.97)	0.20
Yes	1 [Reference]	0.63(0.48, 0.83)	0.49(0.35, 0.70)	0.79(0.56, 1.11)	
diabetes					
No	1 [Reference]	0.67(0.49, 0.92)	0.44(0.30, 0.66)	0.52(0.34, 0.79)	0.01
Yes	1 [Reference]	0.66(0.45, 0.97)	0.62(0.38, 1.03)	1.18(0.73, 1.92)	
PIR					
<1	1 [Reference]	0.52(0.34, 0.78)	0.50(0.31, 0.81)	0.50(0.30, 0.83)	0.05
1–3	1 [Reference]	0.76(0.53, 1.08)	0.47(0.29, 0.76)	0.71(0.44, 1.17)	
>3	1 [Reference]	0.94(0.47, 1.89)	0.80(0.35, 1.86)	1.73(0.74, 4.05)	
BMI (kg/m 2)					
<24.9	1 [Reference]	0.55(0.34, 0.88)	0.40(0.23, 0.70)	0.72(0.40, 1.30)	0.34
25.0–29.9	1 [Reference]	0.52(0.31, 0.87)	0.38(0.19, 0.75)	0.49(0.23, 1.04)	
>30.0	1 [Reference]	0.82(0.57, 1.19)	0.61(0.38, 0.97)	0.95(0.60, 1.50)	
**CVD mortality**					
**Serum sodium**					
sex					
Female	1 [Reference]	0.72(0.31, 1.69)	1.04(0.58, 1.85)	0.78(0.38, 1.60)	0.56
Male	1 [Reference]	0.43(0.16, 1.13)	0.42(0.20, 0.89)	0.92(0.40, 2.12)	
age					
<65	1 [Reference]	0.81(0.34, 1.92)	1.11(0.56, 2.17)	0.51(0.17, 1.50)	0.09
≥65	1 [Reference]	0.64(0.25, 1.63)	0.75(0.41, 1.39)	1.37(0.71, 2.65)	
hypertension					
No	1 [Reference]	0.93(0.25, 3.46)	0.81(0.23, 2.87)	0.77(0.17, 3.47)	0.76
Yes	1 [Reference]	0.53(0.25, 1.13)	0.74(0.46, 1.21)	0.88(0.50, 1.57)	
diabetes					
No	1 [Reference]	1.48(0.64, 3.40)	0.70(0.35, 1.37)	1.38(0.67, 2.84)	0.13
Yes	1 [Reference]	0.35(0.12, 1.02)	0.98(0.52, 1.84)	0.51(0.20, 1.27)	
PIR					
<1	1 [Reference]	0.71(0.29, 1.70)	0.76(0.36, 1.58)	0.57(0.24, 1.36)	0.42
1–3	1 [Reference]	0.63(0.21, 1.89)	0.79(0.40, 1.57)	1.18(0.53, 2.65)	
>3	1 [Reference]	1.04(0.23, 4.71)	0.46(0.14, 1.51)	4.39(1.18, 16.3)	
BMI (kg/m 2)					
<24.9	1 [Reference]	0.72(0.23, 2.24)	0.98(0.40, 2.37)	1.05(0.38, 2.89)	0.71
25.0–29.9	1 [Reference]	1.22(0.36, 4.05)	0.63(0.20, 1.99)	0.85(0.20, 3.71)	
>30.0	1 [Reference]	0.26(0.08, 0.87)	0.83(0.43, 1.61)	0.84(0.39, 1.79)	
**Serum potassium**					
sex					
Female	1 [Reference]	1.19(0.57, 2.50)	1.68(0.84, 3.39)	1.58(0.81, 3.09)	0.79
Male	1 [Reference]	0.58(0.20, 1.65)	0.41(0.13, 1.25)	1.53(0.75, 3.13)	
age					
<65	1 [Reference]	1.09(0.49, 2.42)	1.29(0.57, 2.95)	1.49(0.69, 3.24)	0.77
≥65	1 [Reference]	0.75(0.31, 1.83)	0.89(0.39, 2.01)	1.94(1.01, 3.70)	
hypertension					
No	1 [Reference]	3.92(0.92, 16.7)	0.78(0.12, 5.10)	3.96(0.92, 17.0)	0.18
Yes	1 [Reference]	0.64(0.32, 1.29)	1.10(0.60, 2.01)	1.42(0.85, 2.38)	
diabetes					
No	1 [Reference]	1.05(0.49, 2.28)	0.75(0.32, 1.73)	1.46(0.71, 3.00)	0.64
Yes	1 [Reference]	0.82(0.32, 2.12)	1.22(0.54, 2.75)	1.41(0.71, 2.80)	
PIR					
<1	1 [Reference]	1.21(0.56, 2.63)	1.38(0.59, 3.24)	0.86(0.39, 1.87)	0.15
1–3	1 [Reference]	0.77(0.28, 2.10)	0.77(0.31, 1.90)	2.01(0.98, 4.15)	
>3	1 [Reference]	0.09(0.01, 0.71)	0.28(0.08, 0.98)	12.4(4.19, 36.7)	
BMI (kg/m 2)					
<24.9	1 [Reference]	0.86(0.31, 2.35)	0.65(0.24, 1.78)	1.97(0.83, 4.68)	0.91
25.0–29.9	1 [Reference]	0.57(0.13, 2.50)	0.68(0.13, 3.52)	2.32(0.76, 7.13)	
>30.0	1 [Reference]	0.79(0.32, 1.93)	1.96(0.86, 4.45)	2.20(1.06, 4.55)	
**Serum chlorine**					
sex					
Female	1 [Reference]	0.72(0.39, 1.32)	0.51(0.23, 1.14)	0.83(0.41, 1.69)	0.96
Male	1 [Reference]	0.40(0.19, 0.83)	0.56(0.24, 1.29)	0.95(0.38, 2.37)	
age					
<65	1 [Reference]	0.54(0.28, 1.05)	0.30(0.11, 0.81)	0.57(0.23, 1.43)	0.08
≥65	1 [Reference]	0.63(0.33, 1.20)	0.86(0.41, 1.81)	1.74(0.85, 3.58)	
hypertension					
No	1 [Reference]	0.29(0.08, 1.02)	0.12(0.02, 0.74)	0.59(0.15, 2.30)	0.76
Yes	1 [Reference]	0.59(0.36, 0.97)	0.61(0.33, 1.14)	0.90(0.49, 1.66)	
diabetes					
No	1 [Reference]	0.65(0.33, 1.28)	0.64(0.29, 1.40)	1.02(0.49, 2.12)	0.77
Yes	1 [Reference]	0.57(0.30, 1.08)	0.43(0.17, 1.06)	0.75(0.31, 1.80)	
PIR					
<1	1 [Reference]	0.35(0.16, 0.76)	0.71(0.32, 1.58)	0.56(0.24, 1.33)	0.07
1–3	1 [Reference]	0.73(0.37, 1.43)	0.44(0.17, 1.16)	0.77(0.31, 1.88)	
>3	1 [Reference]	1.25(0.41, 3.87)	0.11(0.01, 0.87)	24.4(6.97, 85.1)	
BMI (kg/m 2)					
<24.9	1 [Reference]	0.47(0.19, 1.15)	0.71(0.26, 1.90)	1.22(0.44, 3.34)	0.74
25.0–29.9	1 [Reference]	0.78(0.24, 2.51)	0.39(0.07, 2.13)	1.86(0.41, 8.43)	
>30.0	1 [Reference]	0.75(0.38, 1.50)	0.64(0.27, 1.49)	1.10(0.49, 2.46)	

Abbreviations:. CI, Confidence Interval, CVD, Cardiovascular disease; PIR, poverty income ratio; BMI, body mass index; WC, waist circumference.

a: Adjusted by age, sex, race/ethnicity, PIR, education, Marital status, smoking status, drinking status, BMI, WC, hypertension, hyperlipidemia, Total cholesterol, stroke, smoking, cancer or malignancy, and diabetes.

There was a significant interaction between serum potassium and hypertension with the risk of all-cause mortality in patients with depression (P = 0.03 for interaction). For the subgroup with hypertension, compared with the reference group (the first quartile), the HR for all-cause mortality in the second, third, and fourth quartile was 0.81(95% CI, 0.57–1.16), 1.21(95% CI, 0.87–1.68), and 1.39(95% CI, 1.04–1.87), respectively. For the subgroup without hypertension, compared with the reference group, the HR of all-cause mortality in the second, third, and fourth quartile was 1.67(95% CI, 0.79–3.55), 2.02(95% CI, 0.98–4.16), and 2.62(95% CI, 1.30–5.31), respectively. No significant interactions were found between serum potassium and any other strata variables with the risk of all-cause mortality. There were no significant interactions between serum potassium and any strata variables with the risk of CVD mortality (Table 3).

There was a significant interaction between serum chlorine and diabetes with the risk of all-cause mortality in patients with depression (P = 0.01 for interaction). For the subgroup with diabetes, compared with the reference group (the first quartile), the HR for all-cause mortality in the second, third, and fourth quartile was 0.66(95% CI, 0.45–0.97), 0.62(95% CI, 0.38–1.03), and 1.18(95% CI, 0.73–1.92), respectively. For the subgroup without diabetes, compared with the reference group, the HR of all-cause mortality in the second, third, and fourth quartile was 0.67(95% CI, 0.49–0.92), 0.44(95% CI, 0.30–0.66), and 0.52(95% CI, 0.34–0.79), respectively. No significant interactions were found between serum chlorine and any other strata variables with the risk of all-cause mortality. There were no significant interactions between serum chlorine and any strata variables with the risk of CVD mortality (Table 3).

## 4.Discussion

### 4.1 Main findings

In this extensive prospective cohort study, we explored the correlation between serum sodium, potassium, and chloride concentrations and the all-cause and CVD mortality rates among adult Americans with depression. Our research indicates that after adjusting for significant potential confounding factors, serum sodium acts as a protective factor for all-cause mortality in the depressed population, meaning that an increase in serum sodium levels is associated with a decrease in all-cause mortality. Serum potassium shows a nonlinear relationship with the all-cause and CVD mortality rates in patients with depression. As serum potassium levels rise, both the all-cause and CVD mortality rates gradually increase. It is noteworthy that compared to the depressed population with comorbid hypertension, those without hypertension exhibit a significantly higher all-cause mortality rate. Serum chloride also demonstrates a nonlinear relationship with the all-cause and CVD mortality rates in patients with depression. A decrease in serum chloride levels is associated with an increase in all-cause mortality. Moreover, compared to the group without diabetes, the group with comorbid diabetes shows a significant increase in all-cause mortality as serum chloride levels rise. This study provides novel evidence for the association between serum concentrations of sodium, potassium, and chloride and the all-cause and CVD mortality rates among the adult depressed population in the United States.

### 4.2 Lower serum sodium levels lead to higher all-cause and CVD mortality in depression

Hyponatremia may exacerbate depressive moods, thereby increasing the all-cause mortality rate in patients with depression. Most cases of hyponatremia develop gradually over more than 48 hours, during which the brain adapts to changes in osmotic pressure, but neuronal excitability may change accordingly, affecting the severity of depression [26,27]. Research by Smith JA et al. indicates that when serum sodium levels rise, there is an increase in the paraventricular nucleus (PVN) and the supraoptic nucleus (SON) near amygdala (AMYG), along with a change in their excitability, which produces an anxiolytic effect [28]. Some scholars have also confirmed that hyponatremia may intensify depressive moods, potentially leading to heightened activation of amygdala and hypothalamus (HYPO), and a reduction in neural connectivity between HYPO and the orbitofrontal cortex (OFC), as well as between AMYG and HYPO (HIPP); this reduction in connectivity enhances the cortisol response, resulting in decreased cardiac parasympathetic activity [29–34]. Depressive moods also affect the interaction between the hypothalamic-pituitary-adrenal (HPA) axis and AMYG, increasing the incidence and mortality risk of CVD. Furthermore, research by Goldstein P et al. has found that sodium intake is inversely correlated with depression [12]. The study by Liu D further points out that increasing salt intake can stimulate appetite and improve nutritional status, thereby reducing mortality in certain populations [35,36].

Hyponatremia increases the risk of fractures, thereby elevating the all-cause mortality rate in patients with depression [37,38]. Furthermore, Van Poelgeest EP has indicated that neuropsychiatric changes in certain brain regions of patients with depression lead to psychomotor retardation, ataxia, gait and balance abnormalities, sleep disturbances, and attention deficits [39]. Depression also causes bone loss and reduced bone density, which may share genetic factors with fractures, such as SGIP1, significantly increasing the risk of fractures. Moreover, antidepressant medications may induce orthostatic hypotension, cardiac conduction and rhythm disorders, and movement disorders, which further increase the risk of fractures even death [40–45]. Our baseline characteristics suggest that lower serum sodium levels are associated with lower serum calcium levels which might give rise to a higher risk of motality.

Decreased serum sodium levels may induce oxidative stress and inflammatory responses, contributing to an increased all-cause mortality rate in patients with depression. Research by Barsony J et al. suggests that hyponatremia may lead to bone loss through mechanisms involving oxidative stress, causing mitochondria to swell and expand, thereby weakening the myocardial cells’ resistance to oxidative stress, which can lead to heart failure and death [46–48]. Marroncini G’s research indicates that hyponatremia-induced oxidative stress activates Kupffer cells in the lumen of the liver sinusoids, leading to the production of inflammatory cytokines and other factors, which may result in liver fibrosis [49]. Concurrently, the lifestyle or behaviors of patients with depression may excessively activate the hypothalamic-pituitary-adrenal (HPA) axis, leading to immune system dysregulation, stimulating the production of ROS as well as pro-inflammatory cytokines (such as IL-1β, TNF-α), which can cause endothelial dysfunction and induce the formation of atherosclerotic plaques [50,51].

### 4.3 Elevated serum potassium leads to increased CVD and all-cause mortality in depression

Our research indicates that serum potassium levels in patients with depression exhibit a nonlinear correlation with all-cause and CVD mortality rates. Coentre R’s study has found that hypokalemia is associated with the onset and exacerbation of depressive episodes [52]. Besides, a cohort study in Japan also found the deficiencies in potassium intake may be related to depressive symptoms, especially in women [53]. A prospective study featured on the relationship between depressive symptoms and mortality risk in chronic kidney disease revealed the link between low potassium level and depressive symptoms [54]. Our subgroup analysis further reveals that, compared to patients with depression who also have hypertension, those without hypertension have a significantly higher all-cause mortality rate. Zhao Z’s research suggests that antidepressant medications, particularly SSRIs and serotonin-norepinephrine reuptake inhibitors (SNRIs), may affect the levels of serum potassium and magnesium [55]. Such effects could lead to electrolyte imbalances, thereby increasing the risk of arrhythmias and even sudden death. The findings of Vieweg WV et al. also support this notion, as they discovered that tricyclic antidepressants may similarly lead to adverse outcomes [56].

### 4.4 Decreased serum chlorine leads to increased CVD and all-cause mortality in depression

Serum chloride plays a pivotal role in physiological processes such as maintaining osmotic pressure, supporting muscle activity, and regulating blood pressure, yet it is often overlooked in clinical practice [57–59]. It is noteworthy that serum chloride and sodium levels tend to vary in tandem [60]. Research by Liang H et al. has found a positive correlation between serum chloride and the severity of anxiety and depression (R = 0.246, p-value < 0.05), a finding that contradicts the notion that hyponatremia may exacerbate depressive symptoms. The study by Zhang Ying et al. has demonstrated that serum chloride is independently associated with all-cause mortality (HR: 0.922; 95% CI: 0.887–0.958; p < 0.001), supporting our findings. Multiple studies have confirmed that hypochloremia is associated with increased mortality in a variety of diseases, including cardiovascular diseases, chronic kidney disease (CKD), pulmonary diseases, liver diseases, and rectal cancer [61–63]. Research by Radulović B indicates that patients with hypochloremia are more prone to hyponatremia, significantly increasing the risk of death in patients with congestive heart failure, possibly because hypochloremia activates the lysine kinase family, enhancing resistance to diuretics [64,65]. Hypochloremia may also increase the release of renin by reducing chloride transport in the macula densa and affect the activity of myocardial chloride ion channels, inducing arrhythmias and myocardial remodeling, thereby exacerbating the cardiac burden [66,67].

## 5. Conclusion

Our research indicates that elevated serum sodium levels may lead to a reduction in all-cause mortality among patients with depression; however, when the economic level is higher, an increase in serum sodium significantly raises the all-cause mortality rate in patients with depression; an increase in serum potassium may lead to an increase in all-cause and CVD mortality rates in patients with depression, with those without hypertension having a significantly higher all-cause mortality rate than those with hypertension. An increase in serum chloride may lead to a reduction in all-cause mortality in patients with depression; however, when comorbid with diabetes, an increase in serum chloride significantly raises the all-cause mortality rate in patients with depression.

## 6. Strengths and limitations

Our study has several strengths. This paper represents the largest study on the levels of serum sodium, potassium, and chloride in patients with depression and all-cause and CVD mortality, taking into account a variety of potential confounding factors. Furthermore, the analysis is based on a nationally representative sample of adult patients with depression in the United States, which aids in the generalizability of the study results. Our study also has some limitations. First, serum electrolyte levels are based on a single serum measurement, which may not accurately reflect long-term status. Second, covariates collected at baseline may change over time, which could attenuate the true association between serum potassium, sodium, and chloride levels and the mortality of patients with depression. Third, since our categorization of serum potassium, sodium, and chloride is based on the quartiles of the study population, our results may not be comparable with other studies using different cut points. Fourth, it is not possible to completely rule out residual or unknown confounding factors.
